# Acute Pericarditis Hiding an Esophageal Perforation

**DOI:** 10.7759/cureus.32608

**Published:** 2022-12-16

**Authors:** Catarina Osório, Lucia Carvalho, Ana Marta Pereira, Mário Nora, Marta Guimarães

**Affiliations:** 1 General Surgery, Centro Hospitalar de Entre Douro e Vouga, Santa Maria da Feira, PRT

**Keywords:** pneumomediastinum, esophageal exclusion, foreign body ingestion, acute pericarditis, esophageal perforation

## Abstract

Esophageal perforations due to foreign body ingestion are uncommon; however, they can be associated with extremely high mortality rate. The most dreadful complication of this entity is the infection of adjacent structures, namely, the mediastinum and the pericardium. A prompt diagnosis and a precocious start of treatment are essential to the prognosis. Thus, a high degree of suspicion is required, especially in older patients. Therapeutic options are highly variable, depend on several factors, and should be individualized to every patient and their clinical status. Surgical treatment with esophageal exclusion and diversion, in extreme circumstances, is mandatory to control the infection source site and prevent further contamination. We report a case of esophageal perforation, presenting 48 hours after onset, that led to multifactorial shock (septic and cardiogenic) due to pericarditis with pericardial and pleural effusion.

## Introduction

Esophageal perforations due to foreign body ingestion are uncommon and often involve dental protheses and fish or chicken bones [1]. Associated injuries of adjacent structures, such as the pericardium, have been reported and impact the clinical course and short-term survival [2]. Pericarditis due to perforation of the thoracic esophagus is extremely rare, and few cases have been reported [3].

The clinical expression of esophageal perforations is highly variable, and in over 60% of the cases, the diagnosis is made 24 hours after symptom onset. In fact, the mortality associated with this entity is still high, between 10% and 20%, and a high index of suspicion is key, since the delay in treatment is the most significant survival predictor [1,2].

Once the diagnosis is established, emergent surgery is the treatment of choice. The main goals of the surgical treatment are to control the source, to prevent further contamination, to provide nutritional support, and to maintain digestive tract continuity [1,4]. We present a case of esophageal perforation that led to pericarditis with pericardial and pleural effusion.

## Case presentation

An 81-year-old female patient presented to the emergency department with a two-day history of dyspnea and epigastric and chest pain. The pain was persistent, with homolateral dorsal irradiation, and associated with vomiting and diarrhea. After careful interrogation, she related the onset of the symptoms to a choking episode with a fish bone, two days before.

**Figure 1:**
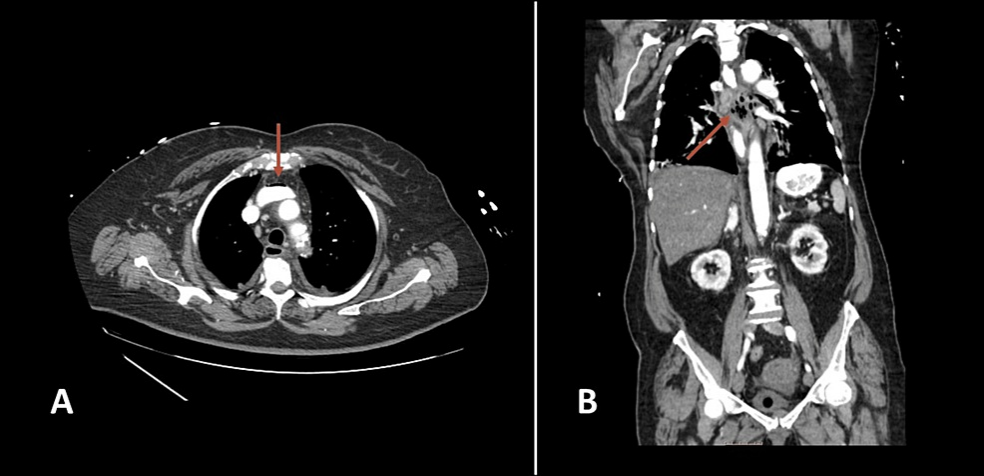
Contrast-enhanced thoracoabdominal CT Axial (A) and coronal (B) sections of contrast-enhanced thoracoabdominal CT demonstrating pneumomediastinum (arrows) CT: computed tomography

**Figure 2:**
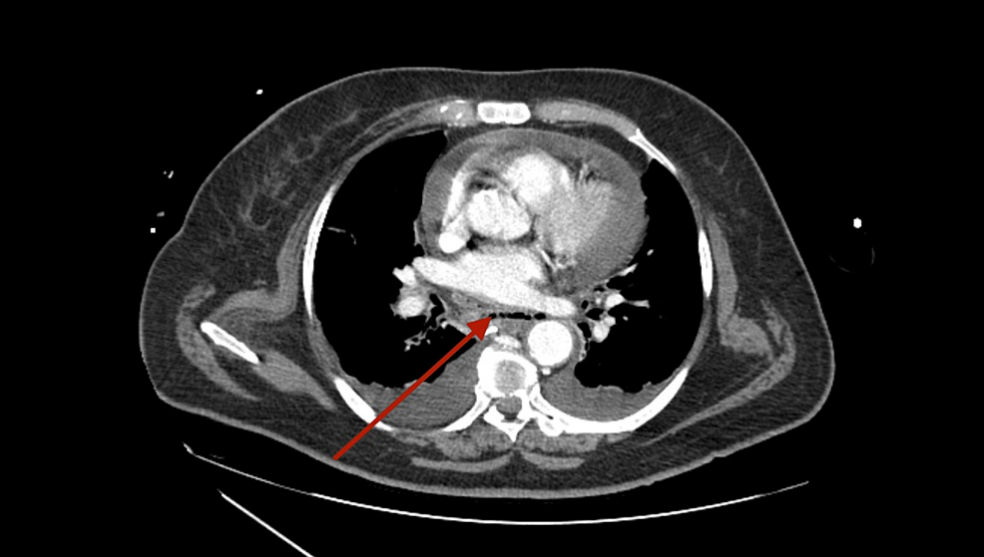
Pre-esophageal infra-carinal collection Contrast-enhanced thoracoabdominal CT showing a pre-esophageal infra-carinal collection with 4 mm maximum anteroposterior thickness and 50 mm in the largest transverse axis, containing liquid and gas bubbles (arrow) suggesting perforation of the esophagus CT: computed tomography

**Figure 3:**
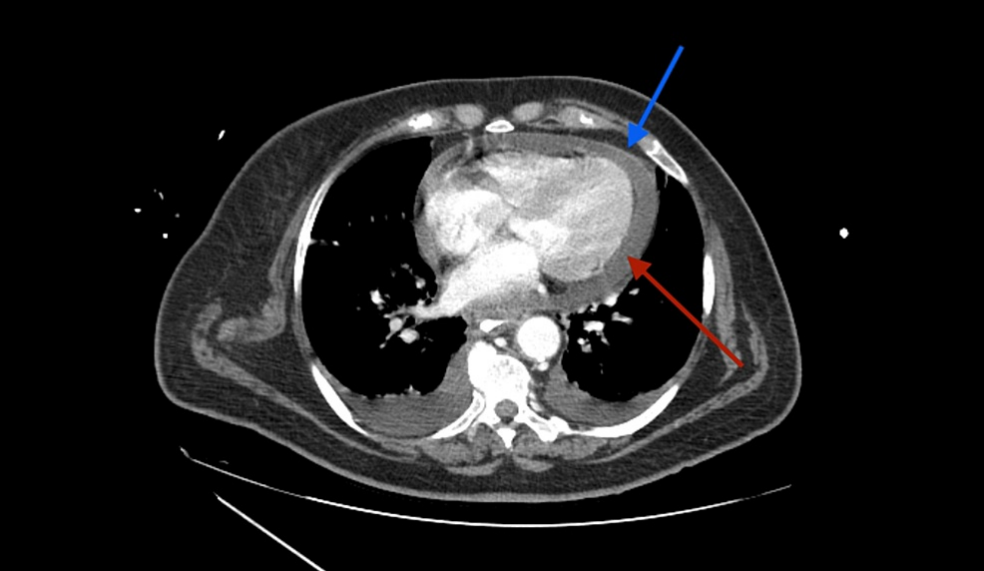
Imagiological signs of acute pericarditis Contrast-enhanced thoracoabdominal CT highlighting a small- to moderate-volume pericardial effusion (red arrow) associated with enhanced pericardial thickening after contrast in favor of pericarditis (blue arrow) CT: computed tomography

On admission, the patient was hypotensive, with polypnea, hypophonesis of cardiac sounds, cold and poorly perfused extremities, and painless abdominal palpation. Arterial blood gas revealed metabolic acidosis with hyperlactacidemia. Due to the established septic shock after fluid challenge, the patient was intubated and began vasopressor support in the emergency room before the etiologic study. Laboratory tests showed leukocytosis (25,400/mm^3^), elevated creatinine levels (1.8 mg/dL), normal amylase and lipase, and elevated levels of C-reactive protein (417 mg/L). An oral and intravenous contrast-enhanced computed tomography (CT) scan of the thorax, abdomen, and pelvis was performed for differential diagnosis and revealed pneumomediastinum (Figure 1A-1B) with a pre-esophageal infra-carinal collection measuring 4×50 mm, containing liquid and gas bubbles but without extravasation of the oral contrast ingested suggesting perforation of the esophagus (Figure 2), as well as bilateral pleural and small- to moderate-volume pericardial effusions associated with enhanced pericardial thickening after contrast in favor of pericarditis (Figure 3) and free intra-abdominal fluid. These findings were consistent with pericarditis secondary to esophageal perforation due to foreign body ingestion. Echo-guided pericardiocentesis was performed, and the pericardial fluid was collected for microbiological and biochemistry analysis; the results showed elevated levels of leukocytes, amylase, and lactate dehydrogenase (LDH) (Table 1).

**Table 1:** Analysis of pericardial fluid after pericardiocentesis LDH: lactate dehydrogenase

Analysis of pericardial fluid	
Red blood cell count (/μL)	200
White blood cell count (/μL)	3,291
Polymorphonuclear leukocytes (%)	91.7
Mononuclear leukocytes (%)	8.3
pH	6.60
Glucose (mg/dL)	<5.0
Total protein (g/dL)	5.0
LDH (unit/L)	1,569
Amylase (unit/L)	440
Albumin (g/dL)	2.9

**Figure 4:**
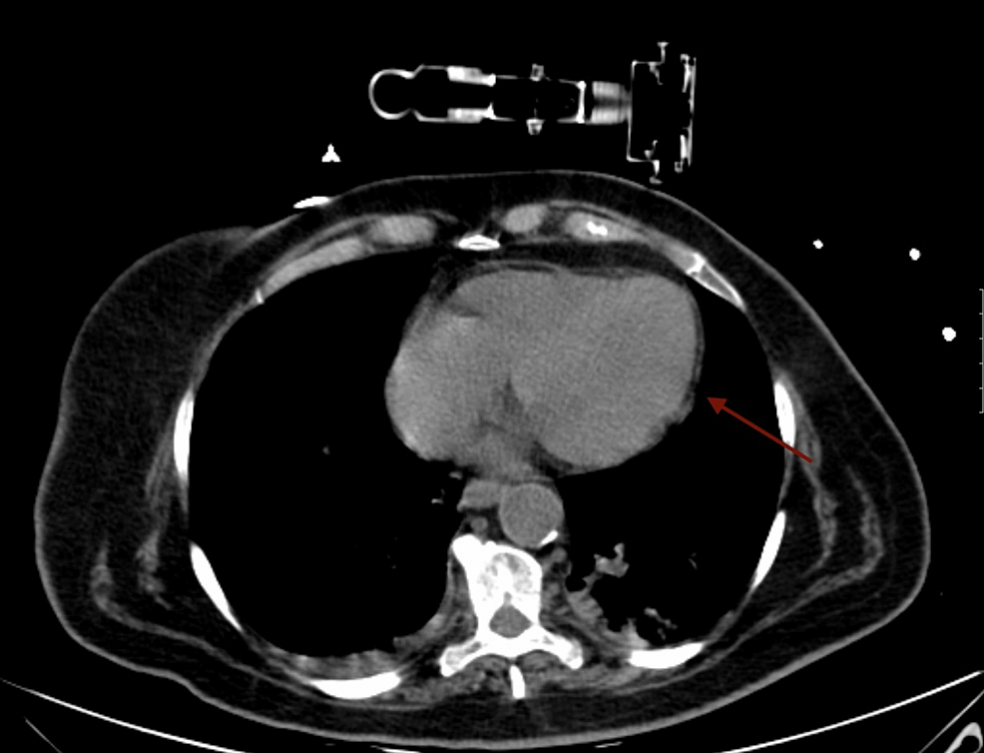
Control CT Control CT revealed the absence of pericardial effusion (arrow) and pneumomediastinum CT: computed tomography

The patient underwent urgent surgery; cervical esophageal exclusion, median laparotomy with transhiatal mediastinal and bilateral pleural drainage, feeding jejunostomy, and echo-guided pericardial drainage with catheter were performed. After the surgical procedure, the initial clinical response was favorable, and a control CT revealed the absence of pericardial effusion and pneumomediastinum (Figure 4).

Unfortunately, despite the prompt initiation of broad-spectrum antibiotics, appropriate local source control of the infection, and invasive organ support in an intensive care unit, the patient died 40 days later due to ventilator-associated pneumonia.

## Discussion

Esophageal perforations involve a myriad of conditions that lead to the disruption of the esophageal wall, with the contamination of the surrounding tissues with digestive contents and consequent infection of deep spaces, such as the mediastinum, that can rapidly progress and evolve to septic shock, in the absence of prompt diagnosis and treatment [2]. Due to its rarity, only isolated cases of pericarditis with effusion after esophageal perforation have been reported (Table 2).

**Table 2:** List of case reports on pericarditis with effusion after esophageal perforation documented in the literature

Authors	Journal	Year
Duman et al.	Turk Kardiyol Dern Ars	2014
Hankins and McLaughlin	J Thorac Cardiovasc Surg	1997
Kupeli and Dogan	J Coll Physicians Surg Pak	2018
Bozer et al.	J Thorac Cardiovasc Surg	1974
Sharland and McCaughan	Ann Thorac Surg	1993
Tsai et al.	Acta Neurol Taiwan	2003
Erdal et al.	J Med Case Rep	2015

Foreign body ingestion is a rare cause of esophageal perforation accounting for 1%-7% of cases [5,6]. The mechanism of perforation appears to be a consequence of both local inflammation and direct pressure necrosis to the wall of the esophagus [5]. The clinical manifestations of esophageal perforation due to foreign body ingestion are usually atypical and are confused with other etiologies such as myocardial infarction, pericarditis, or pulmonary disease, and for this reason, the majority of patients are diagnosed after 24 hours. Therefore, this diagnosis must be always kept in mind in patients presenting with chest pain or dysphagia, particularly in older patients [1]. To avoid delay in diagnosis, a high degree of suspicion is required at presentation. An oral and intravenous contrast-enhanced thoracoabdominal CT is the most sensitive diagnostic tool to detect the perforation; to assess extension to adjacent structures, such as collections or pleural and pericardial effusions; and to guide therapy [2]. A diligent diagnosis is essential as the incidence of complications and mortality rate are dependent on the interval between perforation and the beginning of treatment strategy [7,8]. Delay in diagnosis and treatment is the most important prognostic factor and can result in extremely high morbidity and mortality, reaching up to 26%-45% when treatment is initiated more than 24 hours after the onset [9]. In the case described above, the patient was diagnosed 48 hours after the perforation of the thoracic esophagus with established mediastinitis and pericarditis, which can contribute to the poor prognosis and fatal outcome. Specifically, the presence of pericarditis with pericardial effusion worsened the outcome by aggravating the hypoperfusion and shock status. This is a life-threatening complication of esophageal perforations with a reported incidence of approximately 13% [6].

The treatment focuses on preventing and controlling sepsis and infection and maintaining the continuity of the digestive tract and nutrition. An early intervention, performed within the first 24 hours, offers the most favorable outcomes and success of treatment strategies [4]. The cause of perforation, its location, the degree of contamination, the presence of underlining esophageal disease, and the interval between perforation and diagnosis are critical factors in determining the appropriate treatment approach, although the ideal strategy should be individualized to each patient based on their clinical condition and comorbidities [7,10]. Therapeutic options range from nonoperative management to endoscopic therapies and surgical treatment. Upfront surgical treatment is required in the presence of esophageal perforation with extensive pleural and mediastinal contamination or other complications such as perforation, irretrievable foreign bodies, fistula, or severe bleeding [2]. In the presence of a thoracic esophageal perforation, when a direct repair is not feasible due to hemodynamic instability or extensive esophageal damage, the most appropriate course of action is esophageal exclusion or diversion with the creation of a cervical esophagostomy and feeding jejunostomy [1,10]. In the case described, the hemodynamic state of the patient and the degree of local infection were the bases for the choice of emergent surgical treatment with cervical esophageal exclusion and the drainage of the mediastinum and pericardium. This strategy offered rapid control of infection. The debridement, irrigation, and drainage of mediastinal, pleural, and pericardial space were also important in this case for appropriate local control [4]. Despite the high mortality associated to esophageal perforation, the cause of death of our patient was one of the most common complications associated with mechanical ventilation, affecting up to 40% of these patients [11].

## Conclusions

This case report highlights the importance of prompt diagnosis of esophageal perforations and the need for a high degree of suspicion, particularly in elderly patients with a recent history of foreign body ingestion. The severity of thoracic esophageal perforations is related to the extent of mediastinal and pleural contamination, and although rare, the progression of the infection into the pericardium can be life-threatening. Early recognition and appropriate management are, in fact, the most important prognostic factors and can improve the prognosis associated with this entity. Cervical esophageal exclusion should be the last resort measure, and the selection of this approach should be dictated by the general status of the patient.
